# Outcomes of renal cell carcinoma with associated venous tumor thrombus: experience from a large cohort and short time span in a single center

**DOI:** 10.1186/s12885-021-08508-x

**Published:** 2021-07-02

**Authors:** Zhigang Chen, Feilong Yang, Liyuan Ge, Min Qiu, Zhuo Liu, Cheng Liu, Xiaojun Tian, Shudong Zhang, Lulin Ma

**Affiliations:** 1 Peking University Health Science Centre, No.49 North Garden Road, Haidian District, Beijing, P. R. China; 2 Department of Urology, Peking University Third Hospital, Beijing, 100191 China; 3 Department of Urology, General Hospital of Ningxia Medical University, Yinchuan, 750004 China

**Keywords:** Renal cell carcinoma, Venous tumor thrombus, Thrombectomy, Overall survival, Prognosis

## Abstract

**Background:**

The surgical management and outcomes of renal cell carcinoma (RCC) with venous tumor thrombus (VTT) have been reported in limited sample size, and there remain discrepancies over the factors that influence oncologic outcomes after radical nephrectomy with thrombectomy (RNTE). The aim of the study was to analyze the outcomes of the patients with RCC with VTT in our institution and identify the independent prognostic factors.

**Methods:**

Patients with RCC with VTT were enrolled for the study from February 2015 to December 2018. All patients underwent RNTE. Clinical data were compared using Mann-Whitney U test and the chi-square test for continuous and categorical variables respectively. Survival analysis was estimated using the Kaplan-Meier method. Univariable and multivariable survival analyses were performed using Cox regression model.

**Results:**

121 patients (91 men & 30 women) were identified with a median age of 60 years. VTT level was 0 in 25 patients, I in 20, II in 50, III in 12 and IV in 14. The median follow-up time was 24 months. During the follow-up period, 51 (42%) patients died and 69 (57%) patients experienced recurrence or metastasis. The 3-year and 5-year over-all survival (OS) were 58 and 39%. Among the several factors examined, positive lymph node (*P* = 0.016), metastasis at surgery (*P* = 0.034), tumor necrosis (*P* = 0.023) and sarcomatoid differentiation (*P* < 0.001) were demonstrated as independent significant risk factors on multivariable analysis.

**Conclusion:**

The OS was poor for patients with RCC with VTT. Rather than VTT level, positive lymph node, metastasis at surgery, tumor necrosis and sarcomatoid differentiation were independent prognostic predictors.

## Background

One of the biological characteristics of renal cell carcinoma (RCC) is extending into the venous system, a so-called venous tumor thrombus (VTT). It has been observed that VTT occurs approximately in 4 to 10% patients with RCC [1, 2], including thrombus extending to the renal vein or extending to the inferior vena cava (IVC). Currently, radical nephrectomy with thrombectomy (RNTE) is the standard treatment for RCC with VTT [3]. Though surgical techniques and instruments have improved a lot, RNTE remains the most challenging surgery for urologists, with a relatively high mortality at 2–10% [4–6].

Many centers have reported their experience on the treatment of RCC with VTT, which may guide therapy and patient counseling. Kaplan et al. [7] reviewed their experience from 11 patients with RCC with IVC involvement in 8 years, Wu et al. [8] evaluated the clinical and oncological outcomes in 86 patients with RCC and VTT in 10 years, while Nooromid et al. [9] showed their 15-year experience with RCC with VTT in 37 patients. Though these studies provide a good reference for the treatment of RCC with VTT, two issues also have been identified, which regard a limited sample size and an extensive time span. Obviously, with the rapid development of medical technology, the treatment methods and outcomes may change dramatically during a long-time span. For example, the emerging of targeted therapy offers more choices for RCC with VTT and has been shown to improve survival of RCC with VTT [10]. Considering these findings, it makes sense to analyze a large dataset in a relatively short time span. Accordingly, we hereby present a single center review to understand the outcomes of the 121 patients with RCC and VTT, and to evaluate the significant prognostic factors of overall survival (OS) after RNTE.

## Methods

### Patient selection

**Fig. 1:**
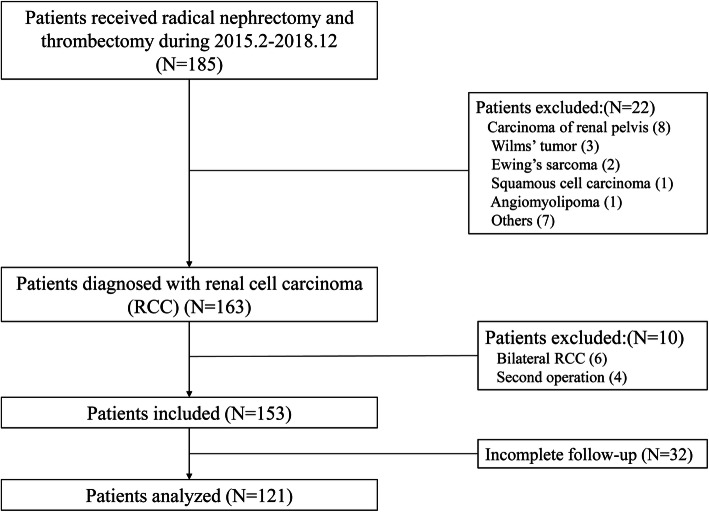
The flow chart of the patient inclusion

The records of 185 patients treated with radical nephrectomy and thrombectomy in our center from February 2015 to December 2018, were retrospectively evaluated. According to the inclusion criteria, only patients whose histopathology diagnosis were RCC were enrolled. The flow chart of patient enrollment was demonstrated in Fig. 1. Before the launching of the study, approval from our Ethics Committee was obtained.

Clinical information, including patient demographics, anaesthesia and surgical situation, as well as cancer and pathological characteristics were obtained from respective medical records. Thrombus levels were classified according to the Mayo Clinic grading system [1]. Pathological variables included histology, TNM stage, Fuhrman nuclear grade, tumor necrosis, sarcomatoid differentiation and metastasis at surgery. TNM staging was performed according to the TNM system of the 2010 American Joint Committee on Cancer (AJCC) [11]. Clavien-Dindo grading system was used to evaluate the post-operative complications [12], and complications of grade ≥ III were defined as major complications.

The VTT above the hepatic veins was defined as high level, while VTT below the hepatic veins was defined as low level in this study. The cut-off values for age, body mass index (BMI), size, tumor thrombus (TT) length, TT width and surgery time were respectively determined based on their mean value. Distant metastasis was confirmed in all patients by imaging examination, including positron emission tomography-computed tomography (PET-CT), contrast enhanced computed tomography (CT) and/or magnetic resonance imaging (MRI), before surgery.

### Follow-up

After surgery, patients were followed up regularly according to the NCCN guidelines [11]. Physical, laboratory and imaging examinations were performed every 3 months for the initial 2 years, semiannually for the next 3 years, and annually thereafter. Follow-up calls were organized to retrieve information of post-operative survival. Recurrence and new metastatic lesions on imaging were defined as disease progression. OS and cancer-specific survival (CSS) was calculated from the time of surgery to the date of death or last follow-up. Progression-free survival (PFS) was defined as the time between the date of surgery and the date of disease progression, death due to disease, or the last follow-up. Patients without the event occurrence were censored at the date of last follow-up.

### Statistical analysis

Patient demographics and clinical characteristics were examined for two distinct subgroups, Mayo 0–II and Mayo III–IV. Differences between these two subgroups were compared using the 2-tailed Mann-Whitney U test for continuous variables, while *χ*2 test for categorical variables. And the Fisher exact test was used, only when the sample size was < 5 per cell. OS, PFS and CSS, as well as 3- and 5-year survival estimates were estimated by using the method of Kaplan-Meier. Prognostic and independent factors were determined by applying univariable and multivariable Cox regression models, respectively. The survival curves, stratified by various clinical parameters, were generated using the Kaplan–Meier method and statistically compared with one another using the log-rank test. All analyses were performed with SPSS version 20.0 (IBM, Armonk, NY), and *P* values < 0.05 were considered statistically significant.

### Results

A total of 185 patients who received RNTE in less than 4 years were identified in our center. After excluding 64 patients who did not meet the inclusion criteria, 121 were available for analysis. Table 1 lists the patient, disease and surgery characteristics. According to the Mayo classification, VTT level was 0 in 25 patients, I in 20, II in 50, III in 12 and IV in 14.

**Table 1:** Patient, cancer and surgery characteristics

Variable	Total	Mayo 0-II	Mayo III-IV	*P* value
N (%)	121	95 (79)	26 (21)	
Median Age (IQR), years	60 (53–67)	59 (53–67)	62 (52.8–68)	0.475
Sex, n (%)				
Male	91 (75)	73 (77)	18 (69)	0.426
Female	30 (25)	22 (23)	8 (31)	
Median BMI (IQR), kg/m^2^	23.3 (21–26)	23 (20.8–26.6)	23.6 (21.6–24.9)	0.714
Tumor side, n (%)				0.912
Left	43 (36)	34 (36)	9 (35)	
Right	78 (64)	61 (64)	17 (65)	
ASA level, n (%)				< 0.001
I + II	95 (79)	84 (88)	11 (42)	
III + IV	26 (21)	11 (12)	15 (58)	
Symptoms at presentation, n (%)				0.011
No symptoms	27 (22)	26 (27)	1 (4.0)	
Symtemic/local symptoms	94 (78)	69 (73)	25 (96)	
Median tumor size (IQR), cm	8 (6–11)	7.3 (5.9–10.6)	8.3 (6–11)	0.246
Clinical T stage, n (%)				< 0.001
T3a	24 (20)	24 (25)	0 (0)	
T3b	48 (40)	44 (46)	4 (15)	
T3c	39 (32)	23 (24)	16 (62)	
T4	10 (8.0)	4 (4.2)	6 (23)	
Surgical methods, n (%)				0.413
Laparoscopic	50 (41)	49 (52)	1 (4.0)	
Open	71 (59)	46 (48)	25 (96)	
Conversion to open, n (%)				0.118
Yes	14 (22)	12 (20)	2 (67)	
No	50 (78)	49 (80)	1 (33)	
Nuclear grade, n (%)				0.254
Grade 1 + 2	49 (40)	41 (43)	8 (31)	
Grade 3 + 4	72 (60)	54 (57)	18 (69)	
Histological type, n (%)				0.236
Clear-cell carcinoma	102 (84)	82 (86)	20 (77)	
Non-clear cell carcinoma	19 (16)	13 (14)	6 (23)	
Cardiopulmonary bypass, n (%)	4 (3.3)	1 (1.1)	3 (12)	0.031
Segmental IVC resection, n (%)	23 (19)	15 (16)	8 (31)	0.109
Median EBL (IQR), mL	700 (200–2550)	500 (200–1600)	2550 (1500–4125)	< 0.001
Median RBC transfusion (IQR), mL	400 (0–1600)	0 (0–1200)	1600 (1200–2500)	< 0.001
Median plasma transfusion (IQR), mL	0 (0–600)	0 (0–400)	700 (350–1200)	< 0.001
Median operative time (IQR), min	338 (242–444)	311 (228–404)	438 (372–520)	< 0.001
Perinephric fat invasion, n (%)	39 (32)	33 (35)	6 (23)	0.260
Sinus fat invasion, n (%)	112 (93)	88 (93)	24 (92)	0.956
Tumor necrosis, n (%)	67 (55)	50 (53)	17 (65)	0.246
Sarcomotoid differentiation, n (%)	28 (23)	20 (21)	8 (31)	0.298
Metastatic disease at resection, n (%)	31 (26)	25 (26)	6 (23)	0.737
Positive lymph node, n (%)	19 (16)	13 (14)	6 (23)	0.243
Adjuvant therapy, n (%)	57 (47)	44 (46)	13 (50)	0.739
ICU admission, n (%)	87 (72)	62 (65)	25 (96)	0.002
Median length of hospital-stay (IQR), days	9 (6–13)	9 (6–11)	12.5 (8.8–16.3)	0.004

*IQR* interquartile range, *BMI* body mass index, *IVC* inferior vena cava, *EBL* estimated blood loss, *RBC* red blood cell, *ICU* intensive care unit

No patients died during the operation. 87 (72%) patients were transferred to the intensive care unit (ICU) after surgery, but most of them (68/87) stayed in ICU for only 1 day. Complications occurred in 50 (41%) patients, of whom 17 had 2 or more complications. Major complications were detected in 17 (14%) patients, including lymphatic leakage infection in 1, acute renal insufficiency in 3, acute cardiac insufficiency in 2, ileus in 2, chylothorax and pleural effusion in 3, left heart failure in 1, deep venous thrombosis in 3, and death in 2. As to the cause of death, one was multiple organ failure associated with the bypass procedures, while another was cardiac arrest.

The histological cell type was clear cell RCC (ccRCC) in 102 (84%) patients and non-clear-cell subtypes in 19 patients (16%). 93% of patients had sinus fat invasion, while 32% had perinephric fat invasion. 16% of patients had lymph node positive disease and 26% of patients had distant metastatic disease at the time of surgery, among which solitary distant metastases were detected in 23 (74%) cases, while multiple distant metastasis was found in 8 (26%) patients. Including these factors into the univariable Cox regression model analysis, lymph node metastasis and distant metastatic disease at surgery were associated with OS (Table 2).

**Table 2:** Univariable and multivariable Cox proportional hazards regression for overall survival

Characteristic	Univariable analysis		Multivariable analysis	
	HR (95% CI)	*P* Value	HR (95% CI)	*P* Value
Sex (female vs male)	1.27 (0.65–2.48)	0.488		
Age (< 59 vs ≧59)	1.20 (0.69–2.07)	0.522		
BMI (< 23.6 vs ≧23.6)	1.07 (0.61–1.88)	0.806		
ASA level (I + II vs III + IV)	1.70 (0.93–3.11)	0.083		
Mayo level (0-II vs III-IV)	1.48 (0.80–2.74)	0.217	1.15 (0.59–2.23)	0.678
Side (left vs right)	1.17 (0.66–2.07)	0.593		
Size (< 8.7 vs ≧8.7)	1.07 (0.61–1.87)	0.816	1.69 (0.92–3.11)	0.088
pN stage (N0 vs N1)	2.33 (1.22–4.48)	0.011	2.30 (1.17–4.55)	0.016
Metastasis at surgery (M0 vs M1)	1.85 (1.05–3.29)	0.035	1.92 (1.05–3.50)	0.034
Pathology (ccRCC vs non-ccRCC)	1.88 (0.80–3.53)	0.051		
Nuclear grade (I + II vs III + IV)	2.22 (1.18–4.18)	0.013	1.92 (0.97–3.84)	0.063
Tumor necrosis (yes vs no)	2.35 (1.30–4.25)	0.005	2.12 (1.11–4.04)	0.023
Sarcomatoid differentiation (yes vs no)	4.40 (2.50–7.73)	< 0.001	3.94 (2.10–7.39)	< 0.001
Sinus fat invasion (yes vs no)	2.69 (0.65–11.08)	0.172		
Renal capsule invasion (yes vs no)	1.70 (0.98–2.94)	0.058		
TT length (< 4.8 vs ≧4.8)	1.30 (0.70–2.40)	0.4		
TT width(< 2.5 vs ≧2.5)	0.85(0.45–1.63)	0.627		
IVC segmental resection (yes vs no)	1.47 (0.77–2.81)	0.244		

*BMI* body mass index, *ASA* American Society of Anesthesiologists, *IVC* inferior vena cava, *TT* tumor thrombus

Compared with the patients with low VTT level, patients with high VTT level had longer median operative time (311 min vs 438 min, *P* < 0.001), more median blood loss (500 ml vs 2550 ml, *P* < 0.001) and longer median length of hospital-stay (9 days vs 12.5 days, *P* = 0.004) (Table 1). Also, patients with high VTT level had a trend toward more frequent cardiopulmonary bypass utilization (12% vs 1.1%, *P* = 0.031) and higher ICU admission rate (96% vs 65%, *P* = 0.002). In addition, progression is more likely to occur in patients with high level VTT (77% vs 52%, *P* = 0.021).

**Fig. 2:**
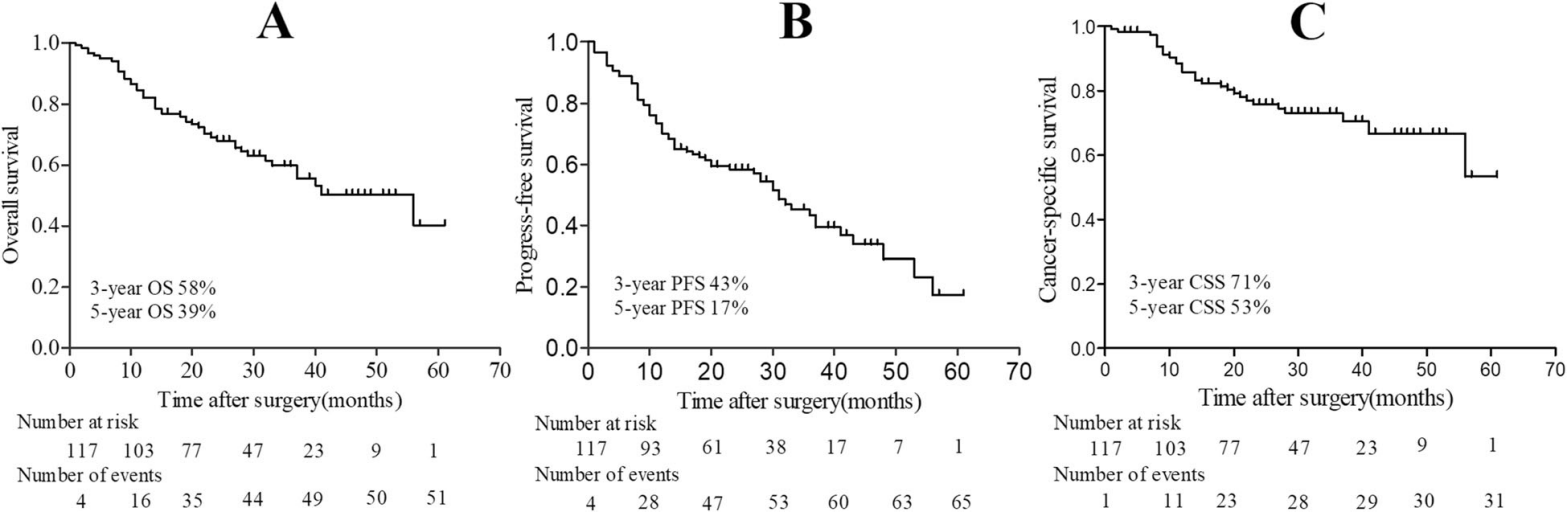
Kaplan–Meier survival curves of survival: **A**, overall survival (OS); **B**, progress-free survival (PFS); **C**, cancer-specific survival (CSS)

The median follow-up time after surgery was 24 months (IQR, 14.5–36 months). During the follow-up period, 57 (47%) patients, including the 31 patients with metastasis at surgery, received postoperative adjuvant therapy, of which, 41 (72%), 14 (25%), 1 (1.8%) and 1 (1.8%) received targeted therapy, cytokine therapy, radiotherapy and targeted therapy plus radiotherapy, respectively. 51 (42%) died from all causes, among which 31 (26%) died from RCC, and 69 (57%) patients experienced progression. The median OS was 41 months (95%CI: 26.6–55.4). As demonstrates in Fig.2, the estimated 3-year OS, PFS and CSS was 58, 43 and 71%; while the 5-year OS, PFS and CSS were 39, 17 and 53%. As to the patients with low VTT level, the 3-year and 5-year OS were 59 and 47%, while it was 48 and 32% for the patients with high VTT level. However, the VTT level was not associated with OS on univariable Cox regression model analysis (Table 2, *P* = 0.217).

**Fig. 3:**
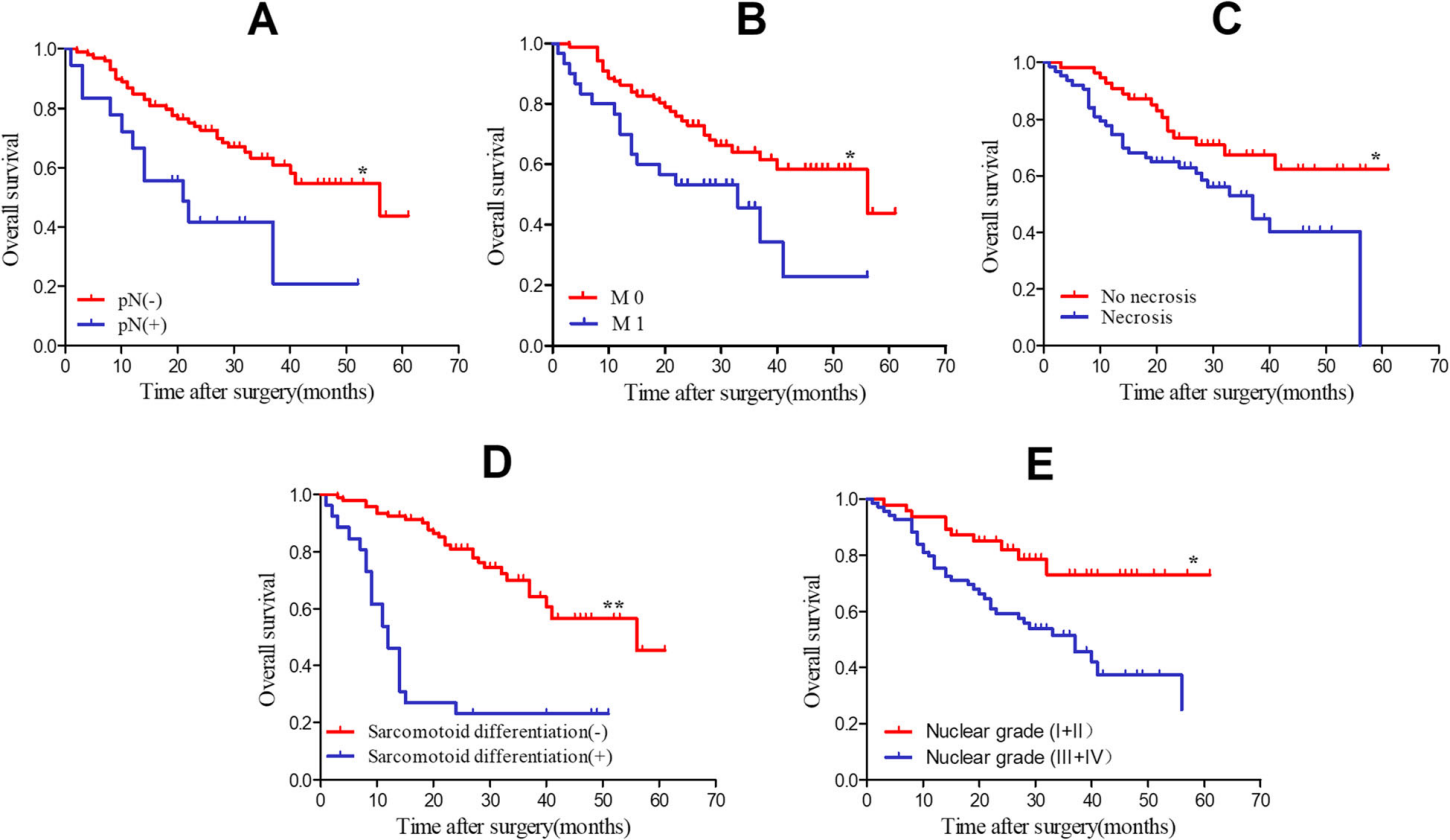
Kaplan–Meier survival curves of overall survival (OS). **A**, stratified by positive or negative lymph node metastasis; **B**, stratified by metastasis at surgery or not; **C**, stratified by tumor necrosis or no tumor necrosis; **D**, stratified by sarcomatoid differentiation or not; **E**, stratified by Nuclear grade (I + II vs III + IV) (*P < 0.05, **P < 0.001)

In terms of independent prognostic factors for patients with RCC with VTT, 5 candidate risk factors were statistically significant in univariable Cox regression model analysis, and the 5 factors were affirmed in Kaplan–Meier survival analysis (Fig. 3). In addition to these 5 factors, another 2 candidate risk factors which may be clinically significant were also further included into a multivariable Cox regression model analysis (Table 2). The result showed that lymph node metastasis (*P* = 0.016, HR 2.30), metastasis at surgery (*P* = 0.034, HR 1.92), tumor necrosis (*P* = 0.023, HR 2.12) and sarcomatoid differentiation (*P* < 0.001, HR 3.94) were identified as independent significant risk factors. Among these four independent significant risk factors, sarcomatoid differentiation is highly correlated with metastasis at surgery (*P* = 0.017), as well as tumor necrosis (*P* = 0.001).

## Discussion

Because VTT is not a common event in RCC patients, it is difficult to collect a large number of RCC patients with VTT in a short time for a single center. Thus, a long-time span and/or a limited sample size are the common problems of the existed studies on RCC with VTT. As one of the largest urology centers in China, we focused on the treatment of RCC with VTT in recent years and attracted a large number of patients. Here we aimed to report the outcomes of 121 patients enrolled in less than 4 years and determine the independent prognostic factors for these patients. To the best of our knowledge, it was rare that such a large number of cases was collected in such a short time span.

The surgical treatment of patients with RCC with VTT remains challenging and technically demanding. Even for experienced urologist, perioperative complications cannot be avoided completely. However, popularization of the multidisciplinary collaboration improves the safety of the patients with RCC with VTT, and decreases mortality [13]. In our cohort, a multidisciplinary team, consisting of a urologist, anesthesiologist, cardiac surgeon, general surgeon, critical care physician and radiologist, offered comprehensive and systematic perioperative plan and management for the patients received RNTE. The complication rate in our study was 41%, which was comparable to prior study [14].

Although surgical techniques and perioperative management have been improved a lot over time, the overall survival of patients with RCC and VTT remains poor, with 5-year OS varied from 37 to 71% [9, 13–18]. This wide variation may result from varied sample size, VTT level, surgical era, tumor pathological classification, comorbidities of patient population and improvements in adjuvant therapy. Consistent with previous studies, the 5-year OS in our study was 39%, which confirmed that RCC with VTT was aggressive with poor prognosis, thus more intensive multimodal therapy should be recommended.

Several patient characteristics and oncologic factors have been identified to be associated with poor oncologic outcomes for RCC with VTT, including BMI, tumor size, tumor necrosis, positive lymph node, metastasis at surgery, Fuhrman grade, and venous invasion [19–22]. However, among these various factors, only positive lymph node, metastasis at surgery, tumor necrosis and sarcomatoid differentiation were determined as independent factors in our study. Though some studies questioned the role of positive lymph node as a prognostic factor on survival [23], this may be contributed to the fact that lymphadenectomy was not routinely performed.

Histological tumor necrosis and sarcomatoid differentiation determine the biological potential and tumor aggressiveness [1]. Many centers had identified tumor necrosis and sarcomatoid differentiation as markers for poor outcomes [8, 19, 24]. Thus, they should be considered as a reference for postoperative adjuvant therapy and design of future clinical trials. Consistent with previous reports, tumor necrosis and sarcomatoid differentiation were identified as independent prognostic factors for OS in present study, the 5-year OS was 37 and 62% for patients with and without tumor necrosis (*P* = 0.005); while for patients with and without sarcomatoid differentiation, it was 21 and 44% (*P* < 0.001).

It remains controversial whether the VTT level could be used as a prognostic predictor in patients with RCC and VTT. Some studies suggested that the level of VTT is associated with long-term survival [25], while others held that the level of VTT is not an independent prognosis predictor [7, 8]. In our study, no correlation between the low and high levels of VTT on OS was detected. Considering the different stages of the VTT in renal vein and in IVC on the basis of AJCC, and some studies reported better survival with only renal vein involvement compared to IVC thrombus [19, 20, 26], we further grouped VTT level I with II, III and IV together, and compared it with Mayo 0. The result was similar that there was no significant difference in outcomes for patients with only renal vein involvement (Mayo 0) compared to IVC thrombus (Mayo I-IV) (*P* = 0.342). Nevertheless, we did find that patients with high level VTT would be more likely experience tumor progress than patients with low level VTT.

Metastasis at surgery was generally reported as a poor prognostic indicator in patients with RCC with VTT [17, 27], with a median survival expectation of less than 1 year. However, some studies found inconsistent outcomes [14, 28], even some patients with metastatic diseases have prolonged survival due to unknown reasons [6, 29]. In our series, the median survival for patients with metastasis was 23 months, which was longer than that in previous report. And metastasis at surgery was identified to be associated with OS in univariable and multivariable Cox regression model analysis. However, considering the fact that the metastasis in our study was mainly confirmed by PET/CT, not pathology, the result of our study needed to be further investigated, though we prefer to believe that metastasis is an independent prognostic factor for RCC patients with VTT.

There are several limitations to this current study. Firstly, this was a retrospective study from a single-institution. So, an inherent selection bias and some confounding factors may not be able to overcome. Secondly, some important variables, such as preoperative laboratory index, thrombus consistency, grafting and reconstruction, status of surgical margin and so on, were not available in the database. Hence, only several commonly available clinicopathologic parameters were included in the univariable and multivariable Cox regression model analyses; however, the results may vary with the different variables included. Thirdly, due to the low number of metastatic patients in our study, clinically non-metastatic and metastatic patients were mixed into the same cohort and analyzed in one group. Fourthly, many important information, such as the time of postoperative adjuvant treatment and side effects of adjuvant therapy, were not fully recorded, which made further analysis unable to be performed. At last, though the influences come from a long-time span were avoided, the median follow-up period of 24 months in this study was inadequate. Therefore, some important information cannot be documented and analyzed.

## Conclusions

We presently conclude that the OS was poor for RCC patients with VTT and rather than VTT level, positive lymph node, metastasis at surgery, tumor necrosis and sarcomatoid differentiation were independent prognostic predictors for RCC patients with VTT.

## Data Availability

The datasets generated during and/or analyzed during the current study are available from the corresponding author on reasonable request.

## Abbreviations

RCC: Renal cell carcinoma
VTT: Venous tumor thrombus
RNTE: Radical nephrectomy with thrombectomy
OS: Over-all survival
CSS: Cancer-specific survival
IVC: Inferior vena cava
AJCC: American Joint Committee on Cancer
MRI: Magnetic resonance imaging
CT: Computed tomography
PFS: Progression-free survival
ICU: Intensive care unit
ccRCC: Clear cell RCC

## References

[1] Blute ML, Leibovich BC, Lohse CM, Cheville JC, Zincke H (2004). The Mayo Clinic experience with surgical management, complications and outcome for patients with renal cell carcinoma and venous tumour thrombus. BJU Int.

[2] Al Otaibi M, Abou Youssif T, Alkhaldi A (2009). Renal cell carcinoma with inferior vena caval extension: impact of tumour extent on surgical outcome. BJU Int.

[3] Martínez-Salamanca JI, Linares E, González J (2014). Lessons learned from the International Renal Cell Carcinoma-Venous Thrombus Consortium (IRCC-VTC). Curr Urol Rep.

[4] Lambert EH, Pierorazio PM, Shabsigh A, Olsson CA, Benson MC, McKiernan JM (2007). Prognostic risk stratification and clinical outcomes in patients undergoing surgical treatment for renal cell carcinoma with vascular tumor thrombus. Urology..

[5] Woodruff DY, Van Veldhuizen P, Muehlebach G (2013). The perioperative management of an inferior vena caval tumor thrombus in patients with renal cell carcinoma. Urol Oncol.

[6] Lardas M, Stewart F, Scrimgeour D, Hofmann F, Marconi L, Dabestani S, Bex A, Volpe A, Canfield SE, Staehler M, Hora M, Powles T, Merseburger AS, Kuczyk MA, Bensalah K, Mulders PFA, Ljungberg B, Lam TBL (2016). Systematic review of surgical management of nonmetastatic renal cell carcinoma with vena caval thrombus. Eur Urol.

[7] Kaplan S, Ekici S, Dogan R (2002). Surgical management of renal cell carcinoma with inferior vena cava tumor thrombus. Am J Surg.

[8] Chen X, Li S, Xu Z, Wang K, Fu D, Liu Q, Wang X, Wu B (2015). Clinical and oncological outcomes in Chinese patients with renal cell carcinoma and venous tumor thrombus extension: single-center experience. World J Surg Oncol.

[9] Nooromid MJ, Ju MH, Havelka GE, Kozlowski JM, Kundu SD, Eskandari MK (2016). Fifteen-year experience with renal cell carcinoma with associated venous tumor thrombus. Surgery..

[10] Qi N, Wu P, Chen J, Li T, Ning X, Wang J, Gong K (2017). Cytoreductive nephrectomy with thrombectomy before targeted therapy improves survival for metastatic renal cell carcinoma with venous tumor thrombus: a single-center experience. World J Surg Oncol.

[11] Edge SB, Compton CC (2010). The American joint committee on Cancer: the 7th edition of the AJCC cancer staging manual and the future of TNM. Ann Surg Oncol.

[12] Mitropoulos D, Artibani W, Graefen M, Remzi M, Rouprêt M, Truss M, European Association of Urology Guidelines Panel (2012). Reporting and grading of complications after urologic surgical procedures: an ad hoc EAU guidelines panel assessment and recommendations. Eur Urol.

[13] Master VA, Ethun CG, Kooby DA, Staley CA, Maithel SK (2018). The value of a cross-discipline team-based approach for resection of renal cell carcinoma with IVC tumor thrombus: a report of a large, contemporary, single-institution experience. J Surg Oncol.

[14] Sweeney P, Wood CG, Pisters LL, Slaton JW, Vaporciyan A, Munsell M, Carpenter S, Putnam J, Swisher SG, Walsh G, Swanson D, Dinney CPN (2003). Surgical management of renal cell carcinoma associated with complex inferior vena caval thrombi. Urol Oncol.

[15] Kalkat MS, Abedin A, Rooney S, Doherty A, Faroqui M, Wallace M, Graham TR (2008). Renal tumours with cavo-atrial extension: surgical management and outcome. Interact Cardiovasc Thorac Surg.

[16] Gu L, Wang Z, Chen L, Ma X, Li H, Nie W, Peng C, Li X, Gao Y, Zhang X (2017). A proposal of post-operative nomogram for overall survival in patients with renal cell carcinoma and venous tumor thrombus. J Surg Oncol.

[17] Xiao X, Zhang L, Chen X, Cui L, Zhu H, Pang D, Yang Y, Wang Q, Wang M, Gao C (2018). Surgical Management of Renal Cell Carcinoma Extending into Venous System: a 20-year experience. Scand J Surg.

[18] Hirono M, Kobayashi M, Tsushima T (2013). Impacts of clinicopathologic and operative factors on short-term and long-term survival in renal cell carcinoma with venous tumor thrombus extension: a multi-institutional retrospective study in Japan. BMC Cancer.

[19] Whitson JM, Reese AC, Meng MV (2013). Population based analysis of survival in patients with renal cell carcinoma and venous tumor thrombus. Urol Oncol.

[20] Staehler G, Brkovic D (2000). The role of radical surgery for renal cell carcinoma with extension into the vena cava. J Urol.

[21] Spiess PE, Kurian T, Lin HY (2012). Preoperative metastatic status, level of thrombus and body mass index predict overall survival in patients undergoing nephrectomy and inferior vena cava thrombectomy. BJU Int.

[22] Armstrong PA, Back MR, Shames ML, Bailey CJ, Kim T, Lawindy SM, Sexton WJ, Spiess PE (2014). Outcomes after inferior vena cava thrombectomy and reconstruction for advanced renal cell carcinoma with tumor thrombus. J Vasc Surg Venous Lymphat Disord.

[23] Sidana A, Goyal J, Aggarwal P, Verma P, Rodriguez R (2012). Determinants of outcomes after resection of renal cell carcinoma with venous involvement. Int Urol Nephrol.

[24] Tornberg SV, Nisen H, Visapää H, Kilpeläinen TP, Järvinen R, Mirtti T, Kantonen I, Simpanen J, Bono P, Taari K, Järvinen P (2016). Outcome of surgery for patients with renal cell carcinoma and tumour thrombus in the era of modern targeted therapy. Scand J Urol.

[25] Martinez-Salamanca JI, Huang WC, Millan I (2011). Prognostic impact of the 2009 UICC/AJCC TNM staging system for renal cell carcinoma with venous extension. Eur Urol.

[26] Wagner B, Patard JJ, Mejean A (2009). Prognostic value of renal vein and inferior vena cava involvement in renal cell carcinoma. Eur Urol.

[27] Tilki D, Nguyen HG, Dall’era MA (2014). Impact of histologic subtype on cancer-specific survival in patients with renal cell carcinoma and tumor thrombus. Eur Urol.

[28] Parekh DJ, Cookson MS, Chapman W (2005). Renal cell carcinoma with renal vein and inferior vena caval involvement: Clinicopathological features, surgical techniques and outcomes. J Urol.

[29] Nesbitt JC, Soltero ER, Dinney CP (1997). Surgical management of renal cell carcinoma with inferior vena cava tumor thrombus. Ann Thorac Surg.

